# Supplementary material for: Iterative improvement in the automatic modular design of robot swarms
Source: PeerJ Comput Sci. 2020 Dec 7;6:e322. doi: 10.7717/peerj-cs.322 (PMC7924708; doi:10.7717/peerj-cs.322)
Supplement: Supplemental Information 3 [file peerj-cs-06-322-s003.zip › argos3/doc/api/standalone/a00456_source.html]

ARGoS: plugins/robots/foot-bot/simulator/footbot\_base\_ground\_rotzonly\_sensor.cpp Source File


- Main Page
- Related Pages
- Namespaces
- Classes
- Files

- File List
- File Members

# plugins/robots/foot-bot/simulator/footbot\_base\_ground\_rotzonly\_sensor.cpp

Go to the documentation of this file.

```
00001 
00007 #include <argos3/core/simulator/simulator.h>
00008 #include <argos3/core/simulator/entity/composable_entity.h>
00009 #include <argos3/core/simulator/entity/embodied_entity.h>
00010 #include <argos3/core/simulator/entity/floor_entity.h>
00011 #include <argos3/plugins/simulator/entities/ground_sensor_equipped_entity.h>
00012 
00013 #include "footbot_base_ground_rotzonly_sensor.h"
00014 
00015 namespace argos {
00016 
00017    /****************************************/
00018    /****************************************/
00019 
00020    static CRange<Real> UNIT(0.0f, 1.0f);
00021 
00022    /****************************************/
00023    /****************************************/
00024 
00025    CFootBotBaseGroundRotZOnlySensor::CFootBotBaseGroundRotZOnlySensor() :
00026       m_pcEmbodiedEntity(NULL),
00027       m_pcFloorEntity(NULL),
00028       m_pcGroundSensorEntity(NULL),
00029       m_pcRNG(NULL),
00030       m_bAddNoise(false),
00031       m_cSpace(CSimulator::GetInstance().GetSpace()) {}
00032 
00033    /****************************************/
00034    /****************************************/
00035 
00036    void CFootBotBaseGroundRotZOnlySensor::SetRobot(CComposableEntity& c_entity) {
00037       m_pcEmbodiedEntity = &(c_entity.GetComponent<CEmbodiedEntity>("body"));
00038       m_pcGroundSensorEntity = &(c_entity.GetComponent<CGroundSensorEquippedEntity>("ground_sensors"));
00039       m_pcGroundSensorEntity->Enable();
00040       m_pcFloorEntity = &m_cSpace.GetFloorEntity();
00041    }
00042 
00043    /****************************************/
00044    /****************************************/
00045 
00046    void CFootBotBaseGroundRotZOnlySensor::Init(TConfigurationNode& t_tree) {
00047       try {
00048          CCI_FootBotBaseGroundSensor::Init(t_tree);
00049          /* Parse noise level */
00050          Real fNoiseLevel = 0.0f;
00051          GetNodeAttributeOrDefault(t_tree, "noise_level", fNoiseLevel, fNoiseLevel);
00052          if(fNoiseLevel < 0.0f) {
00053             THROW_ARGOSEXCEPTION("Can't specify a negative value for the noise level of the foot-bot ground sensor");
00054          }
00055          else if(fNoiseLevel > 0.0f) {
00056             m_bAddNoise = true;
00057             m_cNoiseRange.Set(-fNoiseLevel, fNoiseLevel);
00058             m_pcRNG = CRandom::CreateRNG("argos");
00059          }
00060          m_tReadings.resize(8);
00061       }
00062       catch(CARGoSException& ex) {
00063          THROW_ARGOSEXCEPTION_NESTED("Initialization error in foot-bot rotzonly ground sensor", ex);
00064       }
00065    }
00066 
00067    /****************************************/
00068    /****************************************/
00069 
00070    void CFootBotBaseGroundRotZOnlySensor::Update() {
00071       /*
00072        * We make the assumption that the robot is rotated only wrt to Z
00073        */
00074       /* Get robot position and orientation */
00075       const CVector3& cEntityPos = m_pcEmbodiedEntity->GetOriginAnchor().Position;
00076       const CQuaternion& cEntityRot = m_pcEmbodiedEntity->GetOriginAnchor().Orientation;
00077       CRadians cRotZ, cRotY, cRotX;
00078       cEntityRot.ToEulerAngles(cRotZ, cRotY, cRotX);
00079       /* Set robot center */
00080       CVector2 cCenterPos(cEntityPos.GetX(), cEntityPos.GetY());
00081       /* Position of sensor on the ground after rototranslation */
00082       CVector2 cSensorPos;
00083       /* Go through the sensors */
00084       for(UInt32 i = 0; i < m_tReadings.size(); ++i) {
00085          /* Calculate sensor position on the ground */
00086          cSensorPos = m_pcGroundSensorEntity->GetSensor(i+4).Offset;
00087          cSensorPos.Rotate(cRotZ);
00088          cSensorPos += cCenterPos;
00089          /* Get the color */
00090          const CColor& cColor = m_pcFloorEntity->GetColorAtPoint(cSensorPos.GetX(),
00091                                                                  cSensorPos.GetY());
00092          /* Set the reading */
00093          m_tReadings[i].Value = cColor.ToGrayScale() / 255.0f;
00094          /* Apply noise to the sensor */
00095          if(m_bAddNoise) {
00096             m_tReadings[i].Value += m_pcRNG->Uniform(m_cNoiseRange);
00097          }
00098          /* Set the final reading */
00099          m_tReadings[i].Value = m_tReadings[i].Value < 0.5f ? 0.0f : 1.0f;
00100       }
00101    }
00102 
00103    /****************************************/
00104    /****************************************/
00105 
00106    void CFootBotBaseGroundRotZOnlySensor::Reset() {
00107       for(UInt32 i = 0; i < GetReadings().size(); ++i) {
00108          m_tReadings[i].Value = 0.0f;
00109       }
00110    }
00111 
00112    /****************************************/
00113    /****************************************/
00114 
00115    REGISTER_SENSOR(CFootBotBaseGroundRotZOnlySensor,
00116                    "footbot_base_ground", "rot_z_only",
00117                    "Carlo Pinciroli [ilpincy@gmail.com]",
00118                    "1.0",
00119                    "The foot-bot base ground sensor.",
00120                    "This sensor accesses the foot-bot base ground sensor. For a complete description\n"
00121                    "of its usage, refer to the ci_footbot_base_ground_sensor.h interface. For the XML\n"
00122                    "configuration, refer to the default ground sensor.\n",
00123                    "Usable"
00124                   );
00125 
00126 }
```

---

Generated on 10 Jul 2018 for ARGoS by 
 1.6.1
